# Social Determinants of Health, Maternal Involvement, and Child Development: Direct and Mediated Pathways

**Published:** 2020

**Authors:** Firoozeh SAJEDI, Mahbobeh AHMADI DOULABI, Roshanak VAMEGHI, Alireza AKBARZADEH BAGHBAN, Farzaneh RASHIDI FAKARI, Mohammad Ali MAZAHERI

**Affiliations:** 1 Pediatric Neurorehabilitation Research Center, University of Social Welfare and Rehabilitation Sciences, Tehran, Iran.; 2 Midwifery and Reproductive Health Research Center, Shahid Beheshti University of Medical Sciences, Department of Midwifery and Reproductive Health, School of Nursing and Midwifery, Shahid Beheshti University of Medical Sciences, Tehran, Iran.; 3 Proteomics Research Center,Department of Biostatistics, School of Allied Medical Sciences,Shahid Beheshti University of Medical Sciences, Tehran, Iran.; 4 Midwifery and Reproductive Health Center, School of Nursing and Midwifery, Shahid Beheshti University of Medical Sciences, Tehran, Iran.; 5 Department of Education and Psychology, Faculty of Social Sciences, Shahid Beheshti University, Tehran, Iran.

**Keywords:** Health, Developmental delay, Child development, Mothers, Path analysis

## Abstract

**Objectives:**

In the process of child development, a variety of factors are at play. In this regard, social determinants of health play a determining role in the development and growth of the child. This study aimed to design and test the model for social determinants of health for the development of 36-6o-month-old children in Tehran with the mediation of maternal involvement.

**Materials & Methods:**

This cross-sectional study was conducted among 1067 mothers and their 36-60-month-old children in childcare centers in Tehran, using multistage sampling. Data gathering tools consisted of a demographic questionnaire for mothers and children, a questionnaire on unhealthy behaviors, Ages and Stages Questionnaire, Economic and Social Status Questionnaire, Perceived Social Support Questionnaire, Perceived Stress Questionnaire, Spielberger Anxiety Inventory, Beck Depression Inventory, ENRICH: Marital Satisfaction Scale, and Participation Scale for Parents and Mothers.

**Results:**

Model fit measures were suitable and goodness of fit (RMSEA = 0.031, GFI = 1) was satisfactory. In addition, the results of path analysis indicated that the participation of mothers in the development of children had a direct positive (ß = 0.089) and increasing effect.

**Conclusion:**

Findings indicated that depression, anxiety, stress, and marital satisfaction have both direct and indirect effects on the participation of mothers and child development. Moreover, the model fit measures indicated the utility and high proportionality of the model, as well as the logic of the adjusted relationships of variables based on the conceptual model.

## Introduction

Global strategy and sustainable development goals emphasize children’s health, requiring that each child survive, grow, and reach its development potential (1, 2). In the health system of a country, children's health is of utmost importance since it is the basis of the health of adolescents and future generations who are future parents and employees of a country. As a result, paying attention to children's health has advantageous effects both on the future of the health system of a country and the future functioning of citizens (3). Given that children are vulnerable, their development and growth are relevant to the health system of the society (4). Development in children is a process that improves children’s physical, mental, emotional, and communicative interaction with people and environmental factors. This development process involves the onset of biological characteristics and behaviors stemming from experiential learning in the society (5). 

Five developmental domains relevant in child development include fine motor skills, gross motor skill, communication skills, cognitive or problem-solving, and personal-social domains (6). Furthermore, early in life, healthy development enables children to have a flourishing life in domains such as social, emotional, cognitive, and physical well-being (7), which are negatively affected in case of any delays, leading to irreparable effects in the overall development process (8). Nowadays, at least 200 million children around the world cannot reach their development potential (9). Baker et al. noted that when children do not acquire developmental abilities relevant to their age, they experience a developmental delay or disorder (10); this developmental delay is one of the top priorities of any health and treatment system in most countries (11). In pediatrics, developmental and behavioral problems are of great importance after infections and traumas (12). Additionally, the rate of developmental delays has been reported to be around 7 to 22.4% in different cities of Iran (13,14). 

The development of the child's brain and its neural pathways is affected by biological factors (i.e., nutrition, infectious diseases, genetics and psychological and social factors) adolescence, pregnancy and childhood factors, as well as combined risk factors such as maternal depression (15). Nevertheless, maternal health, stress, poverty, literacy rate, perceived social support, parenting skills, maternity responsibilities along with housing burden can affect the conditions for participation in childcare (16), which in addition to child development is the number one parental responsibility (17). In this regard, the role of the mother starts and continues before and after childhood and contributes to the development of the child by providing a safe and caring environment for the child. The importance of the relationship between the child and his mother has been studied for decades, and empirically, lack of communication with the child has negative consequences in the neonatal and early childhood stages (18), such that participation of parents in the development process leads to greater and better opportunity for care, development, and health (19). This in return leads to increased social, cognitive, motor, and problem-solving skills, adaptive behaviors, emotional regulation, and higher educational performance in childcare centers and schools (20, 21).

Research shows that health inequities start from infancy and early childhood. Accordingly, of all the factors related to child development, social determinants of health such as social class, social deprivation, marginalization, stress, childhood development, unemployment, conditions of work environment, social support, addiction, food, transportation, urbanization, and world migration greatly influence fetal and childhood growth and play a very important role in the development of the child. In fact, this period is affected by various factors, including social factors affecting health, due to the rapid growth and development of the brain in all aspects of life (22-24).

In addition, problems like the mental health of the mother affect both quality and quantity of care and attention given to the child and lead to severe childhood irritation and consequently to learning and behavioral problems (25,26). Also, maternal anxiety contributes to insufficient development of the child by minimizing the mother's abilities and negatively affecting reactions (27). Moreover, the findings of their study showed that a relationship between stressors and mental and emotional problems of parents on the one hand and the emotional problems as well as the delayed growth of children (28, 29). 

Further, poor social and economic level exposes the child to biological and psychological risk factors, as well as diseases, and leads to a change in brain structure and performance, which finally leads to developmental delay and learning deficiency (18,14). According to Emotional Security Theory, any occurrence of marital conflicts between parents compromises children's feelings, safety and the security inside the family and finally leads to parental depression which has negative effects on the child’s developmental process (30).

Consequently, marital satisfaction influences the relationships between parents and children in a way that an inappropriate marital relationship worsens mother-child relationships for various reasons and even increases maternal depression, which in turn, affects the ability of the mother to respond to the child (31). In addition, results of different studies indicate that healthy development of the child is related to the mother's health as well as the social support received from others in the society; lack of this relationship can change mother’s emotional signals, affecting child development on the whole (32, 33).

Parents' involvement in the lives of children can have a permanent impact on children’s health and well-being. Parents interested in the development and welfare of their children contribute more to the development and education of the child. A positive relationship between the mother and the child can result in mother's future involvement in child development. Moreover, it will have a positive effect on other aspects of the child’s life, even in adulthood and his future social relationships (34). Various studies have defined mothers' participation as a solid, firm, organized, and meaningful participation in counseling, planning, implementing, and evaluating programs which assist child development (35).

Given the importance of child development and the significance of developmental delays as well as the necessity to identify the risk factors contributing to this problem in each community, in addition to a small number of studies that consider several risk factors, especially social risk factors and the role of mothers' participation(36), the present study aimed to design and evaluate a model of the social determinants of health for the development of 36-60-month-old children in Tehran with the mediation of maternal involvement. Furthermore, what added to the importance of this study was the fact that it is not known whether child development is affected by the interference of multiple factors or a single independent factor, as well as the fact that this study used path analysis as a suitable statistical method for studying child development and growth.

## Materials & Methods

This research was a cross-sectional path analysis study conducted after obtaining approval of the proposal at the University of Social Welfare and Rehabilitation Sciences in Tehran and the University's Ethics Committee with the code uswr.rec.1393.153. The study population consisted of 1067 mothers and 36 to 60-month-old children attending Tehran's childcare centers. Sampling was done by using the multi-stage sampling technique. First, Tehran was divided into three districts (north, center, and south) and then in each district a number of municipalities were randomly selected. Based on the number of daycare centers in each of the selected districts, several centers (cluster) were selected randomly, and finally in each center convenience sampling was used to select the participants.

Inclusion criteria for the study included all children aged 36-60 months as well as mothers of these children, being Iranian, lack of stressful and disastrous incidents in the past 6 months, children living with both parents, and having no known developmental disorders such as genetic disorders and syndromes. Exclusion criteria consisted of reluctance to continue to participate in the research study. In addition, data gathering tools consisted of a demographic questionnaire for mothers and children, a questionnaire on unhealthy behaviors, Ages and Stages Questionnaire (ASQ), economic and social status, Perceived Social Support Questionnaire, Perceived Stress Questionnaire, Spielberger Anxiety, Beck’s Depression Inventory, ENRICH's Marital Satisfaction Scale, and Participation Scale for Parents and Mothers. 

Economic and Social Status Questionnaire

To assess the economic and social status of the participants, the questionnaire designed by Garmaroudi and Moradi (2010) was used. This questionnaire includes the level of education of the mother and her husband, house size, and the price per square meter of land and facilities (37).

 Spielberger state-trait anxiety inventory

The state-trait anxiety inventory (STAI) includes separate self-assessment scales for measuring state and trait anxiety. The trait anxiety subscale includes 20 items which measure the individual's emotions at the time of filling out the questionnaire, and the state anxiety subscale includes 20 items that assess the general and ordinary emotions of individuals. The minimum score of both trait and state anxiety subscales is 20 and the maximum score is 80 (38). 

Beck’s Depression Inventory

This questionnaire is used to measure the severity of depression which includes 21 items rated on a 4-point scale ranging from 0 (symptom absent) to 3 (severe symptoms). From these 21 items, 2 items are related to affection, 11 items are related to recognition, 2 items are related to apparent behaviors, 5 items are related to somatic symptoms, and 1 item is related to interpersonal meaning. The minimum score is 0 and the maximum is 63. Scores up to 13: None or least depression, 14 to 19: mild depression, 20 to 28: moderate depression, and 29 to 63: severe depression (39).

Perceived Stress Scale (PSS-14)

The questionnaire is used to measure perceived public stress over the past month. The questionnaire consists of 14 items scored on a five-point Likert scale ranging from 0 to 7, with 7 negative items indicating the inability to cope with stress and 7 positive items indicating the person’s good handling of stressors. The lowest score on this scale is 0 and the highest score is 56 (40).

Social Support Appraisals (SS-A) Scale

This questionnaire consists of 23 items measuring how much a person believes in the interest and respect of family, friends, and others. The subscale of the family has 8 items, the subscale of friends has 7 items, and the subscale of the other includes 8 items. In this research, a modified form of social support questionnaire was used; therefore, each of the options, "yes" or "no", according to the content of sentences, was valued zero or one. The highest and lowest possible scores on this scale are 0 and 23 (41, 42).

ENRICH: Marital Satisfaction Scale (EMS)

This scale was used to examine marital satisfaction. It consists of 35 items scored based on a five-point scale ranging from 1 to 5, and four subscales: marital satisfaction, communication, conflict resolution, and idealistic distortion. This questionnaire has 4 distinct scores for each subscale that are calculated for the total number of items for each subscale. In the end, raw scores are converted to percentages (43). 

Ages and Stages Questionnaire (ASQ)

This is a parental reporting tool which examines and identifies the mental conceptions of the state of child development, with an objective and organized evaluation of the first 4 to 60 months of life. This test consists of 19 sets of questionnaires. For each age group, there are 30 questions, which include 6 questions for each of the five domains of communication, large movements, delicate movements, problem-solving ability, and personal-social skills. There are 3 choices for each of the 30 questions: "yes", "sometimes," and "no". The answer "yes" is assigned 10 points, "sometimes" 5 points, and "no" 0 points. After completing the questionnaires, the scores were compared with the predefined cut-off points based on standardization, such that if the child in each of the five domains fails to reach the cut-off score, it means that he has problems in that area, and necessary specialist follow-up will be required to ensure health or the presence of a disorder or illness (44).

Parental involvement Scale 

This scale consists of 32 items scored based on a Likert Scale ranging from 1 to 4. Based on the time limit in the five sections of one week, one month, days spent from this year, during the past months, and in general and item analysis, the items of the study were categorized into behavioral, cognitive, rational, and personal participation subparts (45). 

In this research, the validity of the partnership scale was determined through face validity and content validity. For determining the face validity, a quantitative method was used. For this purpose, 15 mothers meeting the inclusion criteria rated the items of the questionnaire in terms of the degree of difficulty in understanding the words and phrases, the desirable linkage of phrases, and ambiguity, that is, and the probability of misconceptions. Given that the score of the effect of all instrument items was more than 1.5, all items were retained for review in the next stage.

To establish the content validity of the scale, content validity ratio (CVR) and content validity index (CVI) were used. CVR was determined using the views of 10 specialists and faculty members in children's disciplines (N=4), psychologists (N=3), preschool education (N=2) and health (N=1). In two words, the value was smaller than the Lavasheh value (0.6), which required changes and then, according to experts, the coefficient of influence was calculated again.

The score for the content of the index is above 0.79. On the maternity partnership scale, three of the numbers for the items were lower than 0.79, which required changes in the items, and then, according to the experts, they were calculated again. Based on the suggestion of the experts and the results of the narrative content of some questions, the tool was eventually changed into 40 questions.

Internal reliability of the instrument was determined using the Cronbach's alpha coefficient in the three domains of participation (Cronbach's alpha=0.84, 0.85, 0.75), and Cronbach's alpha coefficient for the total tool was equal to 0.90, which was acceptable. Instrument consistency was also achieved using the scores of the two monitors in 14 people with a two-week interval and the ICC calculation was 89%, indicating satisfactory stability.

In this study, maternal involvement values are the scores they obtained in an involvement evaluation scale. This scale consists of 40 items rated on a 4-point Likert scale of (from 1 to 4) and have been set in five time points (one week, one month, days passed from the year, over the past months, and in general) (45).

 An introduction letter was received from the University of Social Welfare and Rehabilitation Sciences and the Welfare Organization of Tehran province, permission was obtained from relevant welfare centers, then the research objectives were explained to the authorities. Thereafter, through a letter, the eligible children's mothers were asked to present to the kindergartens on a specific day and at a specific time. In the presence of mothers, the researcher explained the research objectives and obtained the consent of mothers meeting the entry criteria. The questionnaires pertaining to the demographic characteristics of mothers and children, unhealthy behaviors, ASQ, economic and social status, perceived social support appraisals, perceived stress, Spielberger's anxiety, Beck's depression, ENRICH couple and parental and maternal involvement were given to them. Then, they returned the completed questionnaires four days later. SPSS version 20 was used to analyze the data and descriptive statistics, central tendency indices, and measures of dispersion, and logistic regression were run. In addition, AMOS software version 20 was used to conduct path analysis. The level of significance was considered 0.05 (P <0.05).

In this study, the appropriateness of the model was determined, the simultaneous correlation between economic and social status, perceived social support appraisals, perceived stress, Spielberger's anxiety, Beck's Depression, ENRICH's couple, parental and maternal involvement scales was investigated using path analysis. 

The path of the model with a square and arrow shows causality. However, path analysis was used to examine the appropriate model and cover the percentage of variance and describe the direct, indirect and total effects of each variable on the dependent and logical variables.

## Results

The results of this study showed that the mean age of mothers and fathers was 31.59 ± 5.35 and 36.16 ± 5.80 years, respectively. In terms of level of education, the majority of mothers and fathers had a high school education. In addition, 51.8% of the children were girls, 67.7% of the children were the first child, 52.2% of mothers had only one child, the monthly income of most families ranged from 400 to 900 thousand tomans (about 30 to 130 dollars). The residential living area of the majority of the sample units was smaller than 25 square meters per person, and the majority of families owned residential units.

Table 1 shows the highest developmental delay in the communication domain (N=72, 6.7 %) and the lowest developmental delay in the personality-social domain (N=37, 3.5 %) (Table 1).

The mean values of mothers' socioeconomic status, state anxiety, trait anxiety, severity of depression, severity of stress, social support, marital satisfaction, and maternal involvement were equal to 22.98 ± 6.19, 42.53 ± 10.13, 44.33 ± 9.03, 12.24 ± 9.41, 33.56 ± 7.61, 16.88 ± 3.1, 109.87 ± 14.14, and 94.31 ± 16.04, respectively.

The results of the regression test revealed that the effect of stress, depression, and socioeconomic status on children's development was significant. In fact, for each one-unit increase in the stress score and keeping other variables constant, the possibility of children's natural development decreased by 2.5%; for a one-unit increase in the depression score and keeping other variables constant, the chance of children's natural development was reduced by 2.3%, and desirable socioeconomic status increased the possibility of children's natural development by 62.8% compared to undesirable socioeconomic status. Other structural and intermediate variables did not show such an effect on children's development (Table 2).

The results of regression test showed that for a one-unit increase in the total involvement score, the probabilities of children's total development, natural development in the domain of problem-solving, and development in the domain of fine movements increased by 1.3%, 1.7%, and 2.1%, respectively (Table 3).

The results showed a significant positive correlation between socioeconomic status and child development (r = 0.120, p <0.001) and maternal involvement (r = 0.199, p <0.001).

The results showed that perceived stress had a significant negative correlation with maternal involvement (r = -0.107, p <0.001) and child development (r = -0.143, p <0.001), depression had a significant negative correlation with maternal involvement (r = -0.187, p <0.001) and child development (r = -0.266, p <0.001), and anxiety had a significant negative correlation with marital satisfaction (r = -0.485, p <0.001), maternal involvement (r = -0.309, p <0.001), and child development (r = -0.297, p <0.001). Moreover, the results showed a significant positive correlation between marital satisfaction and maternal involvement (r = 0.263, p <0.001) and child development (r = 0.223, p <0.001) (Table 4).

In path analysis, the effects of socioeconomic status, depression, stress, anxiety, marital satisfaction variables on children's development were examined (Figure 1).

The fitness indices of RMSEA, NFI, CFI, and GFI in the measured models equaled 1, 0.996, 0.993, and 0.03, respectively, with the degree of freedom of 4 and the Chi-square of 8.040. In this model, the criteria were desirable and the model fitness was good.

Comparison of the direct and indirect effects of social determinants of health (structural and intermediate types) and maternal involvement on the level of children's development are shown in Table 5.

## Discussion

In this study, it was sought to investigate the effect of structural and intermediate determinants of social health on the development of 36 to 60-month-old children in Tehran with the mediating role of maternal involvement. 

The prevalence of developmental delay was 16%. The path analysis model showed that socioeconomic status directly affects depression, anxiety, and participation of mothers and indirectly affects the participation of mothers and child development.

In the present study, a significant relationship was found between socioeconomic status and maternal involvement. Socioeconomic status is one of the most important determinants of health and morbidity and is commonly used to describe social inequalities (46). Vellymalay (2012) believes that socioeconomic status has a stronger effect on involvement than other variables pertaining to parents, such as education (47). The results of the current study regarding socioeconomic status are consistent with those of the study carried out by Diamond et al. 2004 (48). In addition, Duncan and Magnuson referred to parental education, family income, family structure, neighborhood quality of life, and parents' social status as the factors influencing involvement (49). To account for the relationship between low involvement and low socioeconomic status, it can be claimed that parents' job limitations cause them to start working in jobs and locations that need commuting long distances from work to home. In addition, they have to work for long hours and get paid less; therefore, their socioeconomic status affects the amounts of time and energy that parents should devote to their children (50). On the other hand, low socioeconomic level is usually associated with parents' low level of education, which itself intensifies the reduced involvement in child development (51).

In the present study, stress, state anxiety, and trait anxiety had a significant relationship with marital satisfaction and maternal involvement. Psychological problems are associated with feeling of disappointment and frustration, discomfort, loss of motivation and hope, reduced self-confidence, pessimism (52), loss of interpersonal relationships, disruptions in social relations, significant reduction of interest in enjoyable affairs, loss of energy, and deficiency in thinking and decision-making (53). The above-mentioned consequences may constitute the factors leading to one's lower involvement in all family and social affairs, including health and education as well as children's development. 

In studies, the influence of mothers' psychological factors on their involvement has been supported (54-56). According to research findings, high levels of maternal stress, which can arise from the stressful environment (57,58), have a negative effect on the mother’s relationships with family members and are inevitably associated with less interaction with the child (57). Similarly, maternal stress affects the parenting role and leads to a decrease in emotional support towards children and, ultimately, it weakens the motherly role (59). In this vein, LaForett and Mendez reported that symptoms of depression in parents are considered as risk factors for the reduction of parental involvement (56). Kohl et al. also referred to the negative effects of maternal depression on parental involvement (60). Psychological disorders, especially symptoms, such as loss of adequate motivation and energy and negative emotions in mothers leads to their lower interaction with their children as well as with their children's learning and teaching activities. By the same token, research has revealed that high levels of maternal stress are inevitably associated with mothers' lower levels of interaction with children (56, 61). Considering the effects of maternal depression on children's development and educational success, one can regard maternal involvement and participation at home, kindergarten, and childcare center as an intermediary between maternal depression and children's learning and educational successes (60).

The path analysis also showed that marital satisfaction had a positive and incremental effect on maternal involvement and that psychological factors also had a positive and incremental effect on maternal involvement by influencing marital satisfaction. The results of a study done by Patrikakou et al. in this domain showed that marital satisfaction was effective in maternal involvement (62). This finding is in line with that of the present study. According to research findings, there is a negative relationship between mental health and marital satisfaction, and the existence of psychological injuries reduces marital satisfaction (63). Studies have shown that marital satisfaction decreases with increase in depression and anxiety (64). Stress also plays an important role in the quality and stability of intimate relations; thus, it can threaten marital satisfaction, marital survival, and duration of marriage (65, 66). In the case of marital dissatisfaction, marital conflicts arise and, thereby, there will be the possibility of disruption of the relationship between parents and the child and this certainly leads to fewer opportunities for learning experiences in children (67). From among the main aspects of family life, parental involvement and support for each other were most effective in performing parental roles and responsibilities. This support and involvement will strengthen the couple in fulfilling their parental responsibilities and the sense of competence in childcare (68). Conflict between parents, as mentioned earlier, may affect the child-parent relationship and compromise children's psychological well-being (69).

With regard to the path of maternal involvement and children's development, the results of path analysis indicated that maternal involvement had a positive and incremental effect on children's development. This means that children's development is enhanced as maternal involvement increases. The effects of parents on children's development are exerted in the form of direct and indirect interactions. In direct interactions, fathers and mothers rear their children and teach them the skills and techniques they need to succeed in life. On the other hand, children interact with the surrounding world through their parents, which is the indirect interactive effect of parents on children's development (70).

Research has indicated that family involvement is positively correlated with the development of children from pre-school education to adulthood (71). Other studies have shown that parental involvement leads to increased cognitive skills, problem-solving skills, higher academic performance, and more interest in kindergarten, childcare center, and school; therefore, child behavioral problems are reduced (21). In fact, parental involvement plays a central and mediating role in beneficial outcomes for children (73).

The development of a model for 36 to 60-month-old children's development with the approach of social determinants of health (structural and intermediate types) with the mediation of maternal involvement has been presented in this study. For sure, this will not be the only and final model. Even if a model fits the existing data, there are still a number of other models that can have a good fit with these data along with other data. 

One of the limitations of the present study is the non-consideration of the role of other intervening factors in maternal involvement and children's development. It is suggested that other studies be carried out on children's development with other models that interact under the mediating role of maternal involvement and social determinants of health.

## In Conclusion

Findings indicated that depression, anxiety, stress, and marital satisfaction have both direct and indirect effects on maternal participation and child development. Moreover, the goodness of fit model does not differ significantly from the conceptual framework that has been designed based on the review of the literature, and the model fit measures indices indicate the utility, high proportionality of the model, and the logic of the adjusted relationships of variables based on the conceptual model.

## References

[1] Every Woman Every Child (2015). The global strategy for women's, children's and adolescent's health (2016-2030): survive, thrive, transform.

[2] UN SDG (2015). United Nations Sustainable Development Goals.

[3] Marmot MG (2006). Social determinants of health. USA.

[4] Moore TG, McDonald M, Carlon L, O'Rourke KJHpi (2015). Early childhood development and the social determinants of health inequities.

[5] Irwin LG, Siddiqi A, Hertzman C (2010). The equalizing power of early child development: from the Commission on Social Determinants of Health to Action. Child Health Educ..

[6] Kliegman RM, Stanton BM, Geme JS, Schor NF (2016). Nelson Textbook of Pediatrics E-Book.

[7] Marmot M, Friel S, Bell R, Houweling TA, Taylor S ( .2008). CoSDoHJT Closing the gap in a generation: health equity through action on the social determinants of health.

[8] Soleimani F, Sajedi F, Akbari SAA (2014). Developmental delay and related factors. Adv. nurs. midwifery..

[9] Grantham-McGregor S, Cheung YB, Cueto S, Glewwe P, Richter L, Strupp B (2007). Developmental potential in the first 5 years for children in developing countries. The lancet..

[10] Baker RC (2001). Pediatric primary care: well-child care.

[11] Vohr BR, O’Shea M, Wright LL (2003). Longitudinal multicenter follow-up of high-risk infants: why,who, when, and what to assess. Semin Perinatol.

[12] Kliegman R, Stanton B, St Geme J, Schor N (2016). Other congenital heart and vascular malformations. Nelson Textbook of Pediatrics 20th ed Philadelphia.

[13] Amir Ali Akbari S, Torabi F, Soleimani F, Alavi Majd H (2011). Correlation between high risk pregnancy and developmental delay in children 4-60 months in Isfahan, Iran 2010-2011. IRJ..

[14] Khabbazkar F, Dolatian M, Soleimani F, Majd HA (2015). The survey of the correlation between domestic violence during pregnancy and after delivery with developmental status of 8-12 month’s infant. Pajoohandeh..

[15] Mohammadi P (2017). Teratogenic Effects of Some Factors on the Human Fetus: A Review study. J Mil Med..

[16] Rahman A, Iqbal Z, Roberts C, Husain N (2009). Cluster randomized trial of a parent‐based intervention to support early development of children in a low‐income country. Child Care Health Dev..

[17] Amini S (2011). Parental involvement in multicultural preschool settings-A challenge for educators. rapport nr.

[18] Dickinson DK, McCabe A (2001). Bringing it all together: The multiple origins, skills, and environmental supports of early literacy. LDRP..

[19] Vaden-Kiernan N, McManus J, Chapman C (2005). Parent and Family Involvement in Education:2002?.

[20] McClelland MM, Acock AC, Morrison FJ (2006). The impact of kindergarten learning-related skills on academic trajectories at the end of elementary school. ECRQ..

[21] Corcoran J, Dattalo P (2006). Parent involvement in treatment for ADHD: A meta-analysis of the published studies. Res Soc Work Pract..

[22] Maggi S, Irwin LG, Siddiqi A, Poureslami I, Hertzman E, Hertzman C (2005). Knowledge network for early child development Analytic and Strategic Review Paper. International Perspectives on Early Child Development.

[23] Richter L (2004). The importance of caregiverchild interactions for the survival and healthy development of young children: A review.

[24] Wilkinson RG, Marmot M (2003). Social determinants of health: the solid facts: World Health Organization.

[25] Piek JP, Dawson L, Smith LM, Gasson N (2008). The role of early fine and gross motor development on later motor and cognitive ability. Hum Mov Sci..

[26] Propper, C LSE STICERD Research Paper No.

[27] Nichols FH, Humenick SS (2000). Childbirth education: practice, research and theory: WB Saunders company.

[28] Herring S, Gray K, Taffe J, Tonge B, Sweeney D, Einfeld S (2006). Behaviour and emotional problems in toddlers with pervasive developmental disorders and developmental delay: associations with parental mental health and family functioning. J Intellect Disabil Res..

[29] Van den Bergh BR, Mulder EJ, Mennes M, Glover V (2005). Antenatal maternal anxiety and stress and the neurobehavioural development of the fetus and child: links and possible mechanisms. A review. Neurosci Biobehav Rev..

[30] Hanington L, Heron J, Stein A, Ramchandani P (2012). Parental depression and child outcomes–is marital conflict the missing link? Child: care, health and development.

[31] Kliegman RM, Behrman RE, Jenson HB, Stanton BM (2007). Nelson textbook of pediatrics e-book: Elsevier Health Sciences.

[32] Greenspan SI, Shanker S (2009). The first idea: How symbols, language, and intelligence evolved from our primate ancestors to modern humans: Da Capo Press.

[33] Alvergne A (2011). Mothers and Others: The Evolutionary Origins of Mutual Understanding. J Biosoc Sci.

[34] Barnard WM (2004). Parent involvement in elementary school and educational attainment. CHILD YOUTH SERV REV..

[35] (2006). Parental Involvement: A Handbook for Childcare Providers. National Children’s Resource Centre, Barnardos: NCsRC (Ireland).

[36] David, S Gochman Health Behavior: Emerging Research Perspectives, 1998.

[37] Garmaroudi G, Moradi A (2010). Instrument designed to measure socioeconomic status in Tehran. Payesh..

[38] Spielberger C (2010). State‐Trait anxiety inventory:Wiley Online Library.

[39] Beck AT, Steer RA, Carbin MG (1988). Psychometric properties of the Beck Depression Inventory: Twenty-five years of evaluation. Clin. Psychol. Rev..

[40] Cohen S, Kamarck T, Mermelstein R (1983). A global measure of perceived stress. J Health Soc Behav.

[41] Rashedi V, Rezaei M, Gharib M, Nabavi SH (2013). Social support for the elderly: Comparison between home and nursing home. Journal of North Khorasan University of Medical Sciences..

[42] Vaux A, Phillips J, Holly L, Thomson B, Williams D, Stewart D (1986). The social support appraisals (SS‐A) scale: Studies of reliability and validity. Am J Community Psychol..

[43] Asoodeh MH, Daneshpour M, Khalili S, Lavasani MG, Shabani MA, Dadras I (2011). Iranian successful family functioning: Communication. Procedia Soc Behav Sci..

[44] Vameghi R, Sajedi F, Mojembari AK, Habiollahi A, Lornezhad HR, Delavar B (2013). Cross-cultural adaptation, validation and standardization of Ages and Stages Questionnaire (ASQ) in Iranian children. Iran J Public Health..

[45] Naseema C (2001). Abdul Gafoor K. Parental Involvement Rating Scale (Pirs).

[46] Genereux M, Auger N, Goneau M, Daniel M (2008). Neighbourhood socioeconomic status, maternal education and adverse birth outcomes among mothers living near highways. J Epidemiol Community Health..

[47] Vellymalay SKN (2012). The impact of parent's socioeconomic status on parental involvement at home: A case study on high achievement Indian students of a Tamil School in Malaysia. IARC..

[48] Diamond JB, Gomez K (2004). African American parents’ educational orientations: The importance of social class and parents’ perceptions of schools. EUS..

[49] Duncan GJ, Magnuson KA Can family socioeconomic resources account for racial and ethnic test score gaps? Futre Child.

[50] Rowan-Kenyon HT, Bell AD, Perna LW (2008). Contextual influences on parental involvement in college going: Variations by socioeconomic class. JHE..

[51] Foley A (2015). African American Parent Perceptions of Barriers to Parental Involvement. 2015. [Dissertation].

[52] Sutter-Dallay A-L, Murray L, Dequae-Merchadou L, Glatigny-Dallay E, Bourgeois M-L, Verdoux H (2011). A prospective longitudinal study of the impact of early postnatal vs chronic maternal depressive symptoms on child development. Eur Psychiatry..

[53] Sadock BJ, Sadock VA (2011). Kaplan and Sadock's synopsis of psychiatry: Behavioral sciences/clinical psychiatry.

[54] Wei H-S, Chen J-K (2014). The relationships between family financial stress, mental health problems, child rearing practice, and school involvement among Taiwanese parents with school-aged children. J Child Fam Stud..

[55] Waanders C, Mendez JL, Downer JT (2007). Parent characteristics, economic stress and neighborhood context as predictors of parent involvement in preschool children's education. J Sch Psychol..

[56] LaForett DR, Mendez JL Parent involvement, parental depression, and program satisfaction among low-income parents participating in a two-generation early childhood education program. Early Educ Dev.2010.

[57] Bor W, Sanders MR, Markie-Dadds C (2002). The effects of the Triple P-Positive Parenting Program on preschool children with cooccurring disruptive behavior and attentional/ hyperactive difficulties. J Abnorm Child Psych..

[58] Moes DR, Frea WD (2000). Using family context to inform intervention planning for the treatment of a child with autism. JPBI..

[59] Lake A, Chan M (2015). Putting science into practice for early child development. The Lancet..

[60] Kohl GO, Lengua LJ, McMahon RJ (2000). Parent involvement in school conceptualizing multiple dimensions and their relations with family and demographic risk factors. J Sch Psychol..

[61] Goodman SH, Gotlib IHJPr (1999). Risk for psychopathology in the children of depressed mothers: a developmental model for understanding mechanisms of transmission.

[62] Patrikakou EN The power of parent involvement: Evidence, ideas, and tools for student success.

[63] Lundblad AM, Hansson K (2005). Relational problems and psychiatric symptoms in couple therapy. Int J Soc Welf..

[64] Shafiee Kandjani A, Azar M, Janbozorgi M, Nouhi S, Hoseini S (2008). Correlation between Women’s Psychopathological Condition and Marital Satisfaction. Pajoohandeh..

[65] Story LB, Repetti R (2006). Daily occupational stressors and marital behavior. JFP..

[66] Randall AK, Bodenmann G (2009). The role of stress on close relationships and marital satisfaction. Clin Psychol Rev..

[67] Iravani M, Sedrpoushan N, Ardakani I (2012). A social work study on violence against women. Management Science Letters..

[68] Solmeyer AR, Feinberg ME (2011). Mother and father adjustment during early parenthood: The roles of infant temperament and coparenting relationship quality. Infant Behav Dev.

[69] Erel O, Burman B (1995). Interrelatedness of marital relations and parent-child relations: a meta-analytic review. Psychol Bull..

[70] Kail RV, Cavanaugh JC (2008). Human development, a life-span view.

[71] Kernan M (2012). Parental Involvement in Early Learning. A review of research, policy and good practice. International Child Development Initiatives (ICDI) Leiden.

[72] Lamb-Parker F, Piotrkowski CS, Baker AJ, Kessler-Sklar S, Clark B, Peay L (2001). Understanding barriers to parent involvement in Head Start: A research-community partnership. Early Child Res Q..

